# SARS-CoV-2 Infection and the Risk of Suicidal and Self-Harm Thoughts and Behaviour: A Systematic Review

**DOI:** 10.1177/07067437221094552

**Published:** 2022-11

**Authors:** Mark Sinyor, Rabia Zaheer, Roger T. Webb, Duleeka Knipe, Emily Eyles, Julian P.T. Higgins, Luke McGuinness, Lena Schmidt, Catherine Macleod-Hall, Dana Dekel, David Gunnell, Ann John

**Affiliations:** 1 Department of Psychiatry, Sunnybrook Health Sciences Centre, Toronto, Canada; 2 Department of Psychiatry, 7938 University of Toronto, Toronto, Canada; 3 Department of Education Services, Centre for Addiction and Mental Health, Toronto, Canada; 4 Division of Psychology and Mental Health, University of Manchester, Manchester, UK; 5 National Institute for Health Research Greater Manchester Patient Safety Translational Research Centre, Manchester, UK; 6 Population Health Sciences, Bristol Medical School, 1980 University of Bristol, Bristol, UK; 7 The National Institute of Health and Care Research Applied Research Collaboration West (NIHR ARC West), University Hospitals Bristol NHS Foundation Trust, Bristol, UK; 8 The National Institute of Health and Care Research Biomedical Research Centre, University Hospitals Bristol NHS Foundation Trust and the University of Bristol, Bristol, UK; 9 Sciome LLC, Research Triangle Park, NC, USA; 10 Population Data Science, 7759 Swansea University, Swansea, UK; 11 Public Health Wales NHS Trust, Wales, UK

**Keywords:** systematic review, SARS-CoV-2, COVID-19, suicide, self-harm

## Abstract

**Objective:**

The COVID-19 pandemic has had a complex impact on risks of suicide and non-fatal self-harm worldwide with some evidence of increased risk in specific populations including women, young people, and people from ethnic minority backgrounds. This review aims to systematically address whether SARS-CoV-2 infection and/or COVID-19 disease confer elevated risk directly.

**Method:**

As part of a larger Living Systematic Review examining self-harm and suicide during the pandemic, automated daily searches using a broad list of keywords were performed on a comprehensive set of databases with data from relevant articles published between January 1, 2020 and July 18, 2021. Eligibility criteria for our present review included studies investigating suicide and/or self-harm in people infected with SARS-CoV-2 with or without manifestations of COVID-19 disease with a comparator group who did not have infection or disease. Suicidal and self-harm thoughts and behaviour (STBs) were outcomes of interest. Studies were excluded if they reported data for people who only had potential infection/disease without a confirmed exposure, clinical/molecular diagnosis or self-report of a positive SARS-CoV-2 test result. Studies of news reports, treatment studies, and ecological studies examining rates of both SARS-CoV-2 infections and suicide/self-harm rates across a region were also excluded.

**Results:**

We identified 12 studies examining STBs in nine distinct samples of people with SARS-CoV-2. These studies, which investigated STBs in the general population and in subpopulations, including healthcare workers, generally found positive associations between SARS-CoV-2 infection and/or COVID-19 disease and subsequent suicidal/self-harm thoughts and suicidal/self-harm behaviour.

**Conclusions:**

This review identified some evidence that infection with SARS-CoV-2 and/or COVID-19 disease may be associated with increased risks for suicidal and self-harm thoughts and behaviours but a causal link cannot be inferred. Further research with longer follow-up periods is required to confirm these findings and to establish whether these associations are causal.

## Abrégé

**Objectif:**

La pandémie de la COVID-19 a eu un effet complexe sur les risques de suicide et l’autodestruction non mortelle dans le monde entier, et certaines données probantes indiquent un risque accru chez des populations spécifiques dont des femmes, des jeunes gens, et des personnes d’antécédents ethniques minoritaires. La présente revue vise à systématiquement aborder si l’infection par SARS-CoV-2 et/ou la maladie de la COVID-19 apportent directement un risque élevé.

**Méthode:**

Dans le cadre d’une revue systématique vivante plus vaste, examinant l’autodestruction et le suicide durant la pandémie, des recherches automatisées quotidiennes à l’aide d’une liste meublée de mots-clés ont été exécutées dans un ensemble complet de bases de données avec des données d’articles pertinents publiés entre le 1er janvier 2020 et le 18 juillet 2021. Les critères d’admissibilité de notre présente revue comprenaient les études investiguant le suicide et/ou l’autodestruction chez les personnes infectées par le SARS-CoV-2 avec ou sans des manifestations de la maladie de la COVID-19 avec un groupe de comparaison qui n’avait ni infection ni maladie. Les pensées et le comportement suicidaires et d’autodestruction (PCS) étaient des résultats d’intérêt. Les études étaient exclues si elles rapportaient des données pour les personnes n’ayant qu’une infection/maladie potentielle sans une exposition confirmée, un diagnostic clinique/moléculaire ou un auto-rapport d’un résultat de test positif au SARS-CoV-2. Les études de reportages de nouvelles, les études de traitements et les études écologiques examinant les taux des infections par SARS-CoV-2 et les taux de suicide/autodestruction dans une région ont aussi été exclues.

**Résultats:**

Nous avons identifié 12 études qui examinaient les PCS dans neuf échantillons distincts de personnes souffrant du SARS-CoV-2. Ces études, qui investiguaient les PCS dans la population générale et les sous-populations, incluaient des travailleurs de la santé, des associations généralement trouvées positives entre l’infection par SARS-CoV-2 et/ou la maladie de la COVID-19 et les pensées suicidaires/d’autodestruction subséquentes et le comportement suicidaire/d’autodestruction.

**Conclusions:**

La présente revue a identifié certaines données probantes voulant que l’infection par SARS-CoV-2 et/ou la maladie de la COVID-19 peuvent être associées à des risques accrus de pensées et de comportements suicidaires et d’autodestruction mais un lien causal ne peut être déduit. Il faut plus de recherche avec des périodes de suivi plus longues pour confirmer ces résultats et établir si ces associations sont causales.

## Introduction

Pandemics may heighten risks of dying by suicide and of non-fatal self-harm^1^ and concerns have been raised about the potential short- and long-term effects of the COVID-19 pandemic on rates of these outcomes.^2,3^ A study examining the early months of the pandemic across 21 high- and upper-middle income countries found that suicide rates were generally stable or decreased.^4^ However, that finding has not been uniformly observed across populations with some evidence for increased rates of death by suicide and suicide attempts in groups that may be experiencing greater stress during the pandemic including women, young people, and people from ethnic minority backgrounds.^5–7^

COVID-19 has resulted in a myriad of potential stressors that may affect risk of outcomes related to suicide and self-harm in individuals and groups. Notably, contraction of the infectious disease itself may be an important source of stress. As yet, there have been no systematic reviews of the impact of SARS-CoV-2 infection or COVID-19 on suicide and self-harm related outcomes. This study aims to address that gap. It is part of a larger “living” systematic review aimed at characterizing the impact of the COVID-19 pandemic on suicide and self-harm outcomes.^8,9^

Our primary aim was to explore whether SARS-CoV-2 infection, with or without manifestations of COVID-19 disease, is associated with suicide and self-harm related outcomes both in general and in specific populations.

## Methods

The current systematic review is nested within a larger registered living systematic review (PROSPERO ID CRD42020183326; registered on 1st May 2020; https://www.crd.york.ac.uk/PROSPERO/display_record.php?RecordID=183326). Through that review, we conducted automated daily searches using a broad list of keywords on: PubMed; Scopus; medRxiv, PsyArXiv; SocArXiv; bioRxiv; the COVID-19 Open Research Dataset (CORD-19) by Semantic Scholar and the Allen Institute for AI, and the WHO COVID-19 database (see Supplemental Appendix A) to capture studies that have reported findings on suicidal or self-harm thoughts, death by suicide and/or acts of self-harm during the COVID-19 pandemic.^8,9^ For the purposes of this review, “self-harm” was defined as the intentional, non-fatal act of injuring or poisoning oneself regardless of motivation and thus captures both non-suicidal self-injury and suicide attempts.

This systematic review was conducted in accordance with established principles for systematic reviews and PRISMA guidelines.^10,11^ We undertook a two-stage screening process to identify eligible articles that were published between January 1, 2020 and July 18, 2021 that contained any empirical data on suicide and/or self-harm outcomes in people infected with SARS-CoV-2 with or without manifestations of COVID-19 disease. Two authors independently screened article titles and abstracts identifying articles that might contain results on suicide and/or self-harm outcomes and SARS COV-2 status. Full-text articles were subsequently read in detail by one investigator (RZ) who identified articles that were clearly eligible or ineligible according to pre-specified inclusion and exclusion criteria (see below). If articles were not written in English, Google Translate was used to facilitate article screening and, where applicable, data abstraction. Regular meetings were held with the primary investigator (MS) to review in detail articles for which there was any ambiguity regarding eligibility. In these instances, MS and RZ made a joint final determination regarding whether to include or exclude each article.

We deemed studies eligible for inclusion if they included participants who (a) had been diagnosed with SARS-CoV-2 through molecular testing, (b) had been diagnosed with COVID-19 disease through a physician assessment, (c) exhibited physical symptoms consistent with COVID-19 disease following a confirmed exposure to SARS-CoV-2, or (d) self-reported that they had been infected with SARS-CoV-2 (note that we placed no constraints on the timing of infection; i.e. people with current active illness, in recovery, or unspecified were all allowed, although this information was captured where available). To provide the most comprehensive summary of the published evidence-base, we included studies examining any suicide-related outcome including suicidal and/or self-harm thoughts. However, we made the decision to place greater emphasis on studies focusing on behavioural outcomes. We placed no limit on sample size. Eligible observational study designs for our review included cohort studies, case-control studies, and cross-sectional studies. Studies without a comparator group (i.e., having no SARS-CoV-2 negative group) such as case reports, case series, were not eligible for inclusion in the review but such studies that we identified are presented and discussed in Supplemental Appendix B for completeness (please see Supplemental Appendix B and Supplemental Tables 1 and 2). In cases of missing information, we contacted study authors to request further data and/or analyses for outputs where we identified that such information may have been available and would potentially indicate the article's eligibility for inclusion. In cases of unclear information within articles, we contacted study authors for clarification. Articles made available before peer-review were included in the review given the scarcity of published studies on this topic. Google translation services were used for studies not available in English (please note, only one study not available in English is presented in our Summary of Non-Comparator Studies Section/Supplemental Appendix B and none ultimately met inclusion criteria for this review).

**Table 1.** Suicide and/or Self-Harm Behavior in People With SARS-CoV-2 in Studies With a SARS-CoV-2-Negative Comparator Group.

Authors	Population and setting	Study design	Exposure measure	Sample size of SARS-CoV-2 infected individuals	COVID-19 status (A = Active, R = Recovered, U = Unclear)	Outcome questionnaire used	Outcome time span	Estimates/Findings	Quality assessment overall rating
Iob et al., 2020^12^	Adults (General Population) in UK March 21–April 20, 2020	Cross-sectional	Self-report	198	U	Ad hoc question asking about “self-harming or deliberately hurting”	Past week	The proportion of people engaging in self-harm behaviours was greater in people who reported a COVID-19 diagnosis n = 28(14.2%) compared to those who did not n = 2,146(4.8%) (PR 2.94, 95%CI 2.08–4.15)	Fair
Paul & Fancourt 2021^13^	Adults (General Population) in UK April 1, 2020–May 17, 2021	Cohort	Self-report	Not stated (overall study n = 49,324 participants)	U	Ad hoc question asking about “self-harming or deliberately hurting”	Past week	Having had COVID-19 illness increased risk of self-harm behaviours in the total sample and ages 18–29, 45–59. For all ages/overall sample: unadjusted (OR 1.36, 95%CI 1.21–1.53) adjusted for anxiety symptoms (OR 1.35, 95%CI 1.20–1.53) adjusted for depressive symptoms (OR 1.32, 95%CI 1.17–1.50) adjusted for physical abuse and psychological abuse (OR 1.33, 95%CI 1.17–1.50)	Good
Abel et al., 2021^14^	Primary care patients (aged >16) in UK February 1–December 8, 2020	Cohort	PCR test	Total n = 11,923,105 of whom 2.0% (232780) were recorded as having a positive PCR test	U	Clinical codes from charts for self-harm (including self-poisoning and self-injury episodes of varying suicidal intent)	Not stated	COVID-19 positive patients had higher self-harm incident outcomes: unadjusted HR = 2.09, 95%CI = 1.20–3.64 adjusted HR = 2.21, 95%CI = 1.11–4.39	Good
Mortier et al., 2021^a^^15^	Healthcare workers (from ten hospitals in four autonomous communities in Spain: the Basque Country, Castile and Leon, Catalonia, and the Community of Madrid) May 5–July 23, 2020	Cross-sectional	Self-report (subcategorized as having been hospitalized for COVID-19, having had a positive COVID-19 test or medical diagnosis not requiring hospitalization, and all others)	Having been hospitalized for COVID-19 (n = 55); Positive SARS-CoV-2 test or medical COVID-19 diagnosis (n = 845); No COVID-19 diagnosis (n = 4,264)	U	Modified self-report version of selected items from the Columbia Suicide Severity Rating Scale measuring suicide attempt (“make a suicide attempt [i.e., purposefully hurt yourself with at least some intent to die]”).	Past 30 days	The proportion of suicide attempts was greater in those hospitalized with COVID-19 Diagnosis compared to those with no diagnosis: n = 1(1.8%) vs. 3(0.07%) (PR 25.84, 95%CI 2.73–244.58^b^) There was no difference in proportion of suicide attempts between those with a positive test and/or a medical diagnosis compared to with no diagnosis: n = 2(0.24%) vs. 3(0.07%) (PR 3.36, 95%CI 0.56–20.12^b^)	Good

^a^ This paper combined suicidal thoughts with a plan and suicide attempts. The data presented here on attempts only were provided through correspondence with the authors.

^b^ Calculations conducted using raw data.

**Table 2.** Suicide and/or Self-Harm Thoughts in People With SARS-CoV-2 in Studies With a SARS-CoV-2-Negative Comparator Group.

Authors	Population and setting	Study design	Exposure measure	Sample size of SARS-CoV-2-infected individuals	COVID-19 status (U = Unclear, R = Recovered, A = Active)	Outcome Questionnaire Used	Outcome time span	Estimates/Findings	Quality assessment overall rating
Iob et al., 2020^12^	General Population in UK March 21 –April 20, 2020	Cross-sectional	Self-report	n = 198	U	PHQ-9 Item 9	Past week	The proportion of people reporting self-harm/suicidal thoughts was greater in people who reported a COVID-19 diagnosis n = 66(33.4%) compared to those who did not n = 7918(17.8%) (PR 1.88, 95%CI 1.54–2.29^c^)	Fair
Paul & Fancourt, 2021^13^	Adults (General Population) in UK April 1–May 17, 2021	Cross-sectional	Self-report	Not stated (overall study n = 49,324 participants)	U	PHQ-9 Item 9	Past week	Having had COVID-19 illness increased risk of self-harm thoughts in total sample and at ages 18–29, 45–59. For all ages/overall sample: unadjusted (OR 1.17, 95%CI 1.09–1.25) adjusted for anxiety symptoms (OR 1.11, 95%CI 1.03–1.20) adjusted for depressive symptoms (OR 1.05, 95%CI 0.97–1.13) adjusted for physical abuse and psychological abuse (OR 1.16, 95%CI 1.09–1.25)	Good
Perlis et al., 2021^16^	Adults in USA May 2020–February 2021	Cross-sectional	Self-report of clinician diagnosis or positive COVID-19 test.	n = 5945 (6.5% of the total sample)	U	PHQ-9 Item 9	Past 2 weeks	Those with prior COVID-19 had higher suicide/self-harm thought scores compared to those without: Mean[SD] 1.5[1.06]) vs 0.99[1.09], *p* < .001)	Fair
Raifman et al., 2020^17^	Adults in USA March 31–April 13, 2020	Cross-sectional	Self-report	12	U	PHQ-9 Item 9	Past 2 weeks	Those who had COVID-19 were more likely to report suicidal thoughts compared to those who did not have COVID-19: 66.7% vs 15.9% (PR 4.2, 95%CI 2.8–6.4; aPR 3.5, 95%CI 1.9–6.4)	Good
Elbogen et al., (2021)^a^^18^ Tsai et al., (2021)^a^^19^ Tsai et al., (2021)^a^^20^	Low and middle income (<$75,000) Adults ≥22 years) in USA May-June 2020	Cross-sectional	Self-report	354	U	Mini-International Neuro-psychiatric Interview “Over the last 2 weeks, how often did you consider hurting yourself, felt suicidal, or wish that you were dead?”	Past 2 weeks	Bivariate comparisons: The proportion of people who reported suicidal/self-harm thoughts was greater in those with a positive SARS-CoV-2 test compared to those who did not test n = 322(91%) vs. 938(21%)(PR = 4.30, 95%CI 4.03–4.59^c^) and between those who tested positive compared to those who tested negative n = 322(91%) vs. 804(44%) (PR = 2.05, 95%CI 1.94–2.19^b^). Those who tested negative were also more likely to report suicidal/self-harm thoughts compared to those who did not test 804(44%) vs. 938(21%) (PR 2.09, 95%CI 1.94–2.26^b^) Multivariable logistic regression: Infection with SARS-CoV-2 was associated with suicidal/self-harm thoughts in multivariable logistic regression: unweighted OR 1.91, 95% CI 1.15–3.16 weighted OR 1.95, 95% CI 1.20–3.19	Fair
Mortier et al., 2021^15^	Healthcare workers (from ten hospitals in four autonomous communities in Spain: the Basque Country, Castile and Leon, Catalonia, and the Community of Madrid) May 5–July 23, 2020	Cross-sectional	Self-report (subcategorized as having been hospitalized for COVID-19, having had a positive SARS-CoV-2 test or medical diagnosis not requiring hospitalization, and all others)	Having been hospitalized for COVID-19 (n = 55); Positive SARS-CoV-2 test or medical COVID-19 diagnosis (n = 845); No COVID-19 diagnosis (n = 4264)	U	Modified self-report version of selected items from the Columbia Suicide Severity Rating Scale measuring suicidal thoughts (“wish you were dead or would go to sleep and never wake up” and “have thoughts of killing yourself” with and without a plan)	Past 30 days	Bivariate comparisons: No difference in any suicidal thoughts between those who were hospitalized for COVID-19 compared to those with no history of SARS-CoV-2: 12.6% vs 7.9% (PR = 1.61, 95%CI = 0.80–3.24^b^). Difference between those with SARS-CoV-2 infection/COVID-19 disease without hospitalization compared to those with no history of SARS-CoV-2 : 10.0% vs. 7.9% (PR = 1.27, 95%CI = 1.01–1.59^b^)*** No difference in *passive suicidal ideation only* between those who were hospitalized for COVID-19 compared to those with no history of SARS-CoV-2: 4.5% vs 4.7% (PR 0.77, 95%CI 0.20–3.04^b^) or between those with SARS-CoV-2 infection/COVID-19 disease without hospitalization compared to those with no history of SARS-CoV-2: 5.9% vs 4.7% (PR 1.26, 95%CI 0.93–1.70^b^) No difference in a*ctive ideation without plan or attempt only* between those who were hospitalized for COVID-19 compared to those with no history of SARS-CoV-2: 0.0% vs. 0.8% (PR 1.10, 95%CI 0.07–17.78^c^) and between those with SARS-CoV-2 infection/COVID-19 disease without hospitalization compared to those with no history of SARS-CoV-2: 1.0% vs. 0.8% (PR 1.18, 95%CI 0.55–2.56^c^) Difference in a*ctive ideation with a plan or attempt only* between those who were hospitalized for COVID-19 compared to those with no history of SARS-CoV-2-: 8.1% vs. 2.5% (PR 2.90, 95%CI 1.11–7.58^b^^,^^c^) and no difference between those with SARS-CoV-2 infection/COVID-19 disease without hospitalization compared to those with no history of SARS-CoV-2: 3.2% vs. 2.5% (PR 1.28, 95%CI 0.84–1.97^c^) No differences in any of the above after multivariable logistic regression controlling for demographic, clinical, and work-related factors.	Good
Bruffaerts et al., 2021^21^	Healthcare workers from three professional associations (medical doctors, practicing psychiatrists, and clinical psychologists) and 4 hospitals in Leuven-Brussels-Antwerp, Belgium 6 April–14 July 2020	Cross-sectional	Self-report	551 Infected with SARS-CoV-2 and quarantined (n = 525); Infected with COVID and hospitalized (n = 26)	U	Modified self-report version of selected items from the Columbia Suicide Severity Rating Scale measuring suicidal thoughts (Death wish “*In the past 30 days, did you wish you were dead or would go to sleep and never wake up*?”; Suicidal ideation “*In the past 30 days, did you have thoughts of killing yourself?*”); suicide plan (“*In the past 30 days, did you think about how you might kill yourself [*e.g.*, taking pills, shooting yourself] or work out a plan of how to kill yourself?*”)	Past 30 days	Individual level associations: No association between infected with SARS-CoV-2 and quarantined: Death wish: aOR 0.8, 95% CI 0.4–1.4 Suicidal ideation: aOR 1.0, 95% CI 0.5–2.2 Suicide plan: aOR 1.3, 95% CI 0.6–2.5 Associations between infected with SARS-CoV-2 and hospitalized &: Death wish: aOR 11.8, 95% CI 2.1–67.6 Suicidal ideation: aOR 7.6, 95% CI 1.4–41.5 Suicide plan: aOR 11.6, 95% CI 2.5–52.7 aOR: Adjusted odds ratios accounting for age, gender, profession, social supports as well as clinical and work-related factors. Society-level association: Being hospitalized because of COVID19 infection accounted for a small (<5%) but significant population attributable risk proportion for suicide outcomes	Good
Na et al., 2021^22^	Military veterans who survived COVID-19 in USA 2019–2020	Cross– sectional	Self-report	233	U	Two items adapted from the PHQ-9 Item 9. A positive screen was indicated by a response of “several days,” “more than half the days,” or “nearly every day” to at least one of the following questions: “How often have you been bothered by thoughts that you might be better off dead?” and “How often have you been bothered by thoughts of hurting yourself in some way?”	Past 2 weeks	Veterans with SARS-CoV-2 were more likely to report current suicidal/self-harm ideation in comparison to those who were not infected (12.0% vs 7.6%, PR 1.58, 1.09–2.29^c^)	Fair
Na et al., 2021^23^	Military veterans in USA	Repeat cross-sectional	Self-report	661	U	Two items adapted from the PHQ-9 Item 9. A positive screen was indicated by a response of “several days,” “more than half the days,” or “nearly every day” to at least one of the following questions: “How often have you been bothered by thoughts that you might be better off dead?” and “How often have you been bothered by thoughts of hurting yourself in some way?”	Past 2 weeks	27.3% of veterans who were infected with SARS-CoV-2 reported suicidal/self-harm thoughts compared to 18.2% who were not infected. Those who were infected with SARS-CoV-2 were more likely to report current suicidal/self-harm thoughts (PR 1.50, 95%CI, 1.00 to 2.25^c^) Veterans infected with SARS-CoV-2 who were aged 45–59 and 60 + and those in the lowest quartile of pre-pandemic purpose in life had a higher risk of suicidal/self-harm thoughts (*p* < 0.05).	Good

^a^ Studies used same dataset hence results pooled.

^b^ Calculations conducted using raw data.

^c^ Due to a zero value, 0.5 was added to all cells for computation of the relative risk.^24^.

***Note that the three-way comparison reported in this paper was not significant; however, when we converted to bivariate comparisons in order to calculate odds ratios, we identified a significant difference between those with SARS Cov-2 compared to those with no history of SARS-CoV-2.

We excluded studies that reported only on people with physical symptoms that could relate to COVID-19 disease without a confirmed exposure, clinical/molecular diagnosis or self-report of a positive SARS-CoV-2 test result, people under surveillance for the disease without a confirmed diagnosis, people who were worried about having SARS-CoV-2 infection with either no testing or negative testing, or in cases where eligible and ineligible groups were combined (e.g. confirmed cases of SARS-CoV-2 and those under surveillance for potential exposure). We established an *a priori* criterion for inclusion of studies with data on such a combined group if a high proportion had a confirmed SARS-CoV-2 infection according to a pre-specified threshold (≥80% SARS-CoV-2 confirmed). Given the high potential for bias in information included in media reports, we excluded studies of news reporting about suicide and/or self-harm in those with SARS-CoV-2 infection/COVID-19 disease. We also excluded ecological studies examining the relationship between rates of SARS-CoV-2 infections in a particular region and rates of suicide and/or self-harm outcomes because any associations may reflect other factors such as the impact of public health control measures in high infection rate areas rather than the direct effect of the virus itself. Finally, we excluded studies examining a specific treatment for COVID-19 patients to avoid confounding due to possible treatment effects.

We assessed risk of bias using National Institutes of Health study quality assessment tools.^25^ For many studies, data and analyses examining SARS-CoV-2 infection and suicide/self-harm outcomes were secondary to the primary research question. For example, a study might have examined rates of self-harm across a random sample from the general population with rigorous effect estimates adjusted for potential confounders. The same study might present self-harm outcomes in those with and without SARS-CoV-2 infection but with no relative risk estimates or formal statistical tests presented for that specific comparison. Therefore, a study might simultaneously have a low risk of bias for its primary outcome and yet a high risk of bias for the outcome germane to our review. Thus, our assessments of quality did not focus on the overall quality of each study but rather the quality of the methodology and presentation of data specifically related to the relationship between SARS-CoV-2 infection and suicide/self-harm outcomes. Two investigators (MS and RZ) independently coded each study for quality according to that approach and met to discuss and resolve any discrepancies. Studies rated as being of “poor” quality (i.e., high risk of bias) were excluded.

The aforementioned investigators independently extracted the following data in tabular format from each article: basic details about articles (e.g. name of first author, year of publication), population and setting of research, study design type, how exposure and outcome were measured and over what timespan, suicide and/or self-harm outcome measured, sample size of SARS-CoV-2-infected individuals, COVID-19 status (A = Active, R = Recovered, U = Unclear), all suicide and/or self-harm related results and funding sources (see Supplemental Appendix C for funding sources). Authors subsequently met to discuss and resolve any discrepancies. In terms of specific effect measures, prevalence ratios (PRs) were extracted for each outcome if available (or, if only proportions were available, where possible, we calculated PRs or risk ratios (RRs) for cross-sectional and cohort studies respectively). Note that our calculated prevalence and RRs are unadjusted. If studies presented measures adjusted for potential confounders (e.g. adjusted odds ratios), we preferentially report those. If PRs were not available or could not be calculated for a particular study, we extracted any other methodologically valid effect measures presented (e.g., odds ratios (ORs), hazard ratios (HRs), differences in mean scores on a standardized questionnaire).

## Results

### Suicide and/or Self-Harm Behaviour

**Figure 1.**
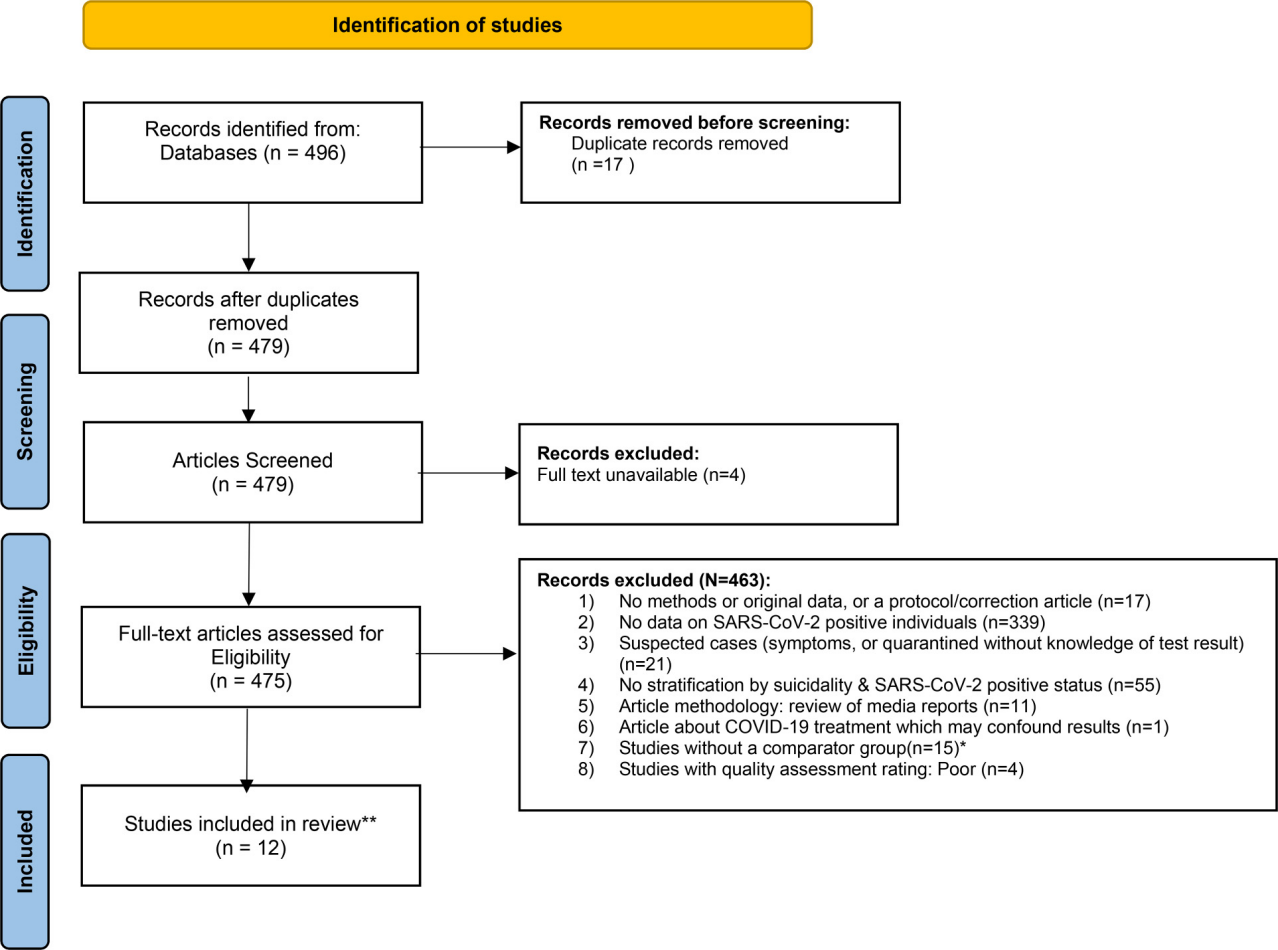
Prisma flow chart.

Four studies examining suicidal and/or self-harm behaviour met the inclusion criteria (Table 1; Figure 1; also see Supplemental Table 3 for details of risk of bias assessment). Three of these were large studies conducted in the United Kingdom with cross-sectional or prospective cohort designs. Two specifically arose from the University College London's, UK (UCL's) COVID-19 Social Study. As part of the Social Study, 44,775 adults were surveyed during the first month of the pandemic of whom 198 reported a SARS-CoV-2 infection. Of these, a larger proportion reported self-harm behaviour within the prior week compared with those without SARS-CoV-2 (14.2% vs. 4.8%; PR 2.94, 95%CI 2.08–4.15).^12^ A follow-up study examined the same outcome over the first year of the pandemic (n = 49,324).^13^ It used fixed-effects regression modeling to predict within-individual change accounting for time-invariant covariates. This approach identified that risk of self-harm behaviour was increased in those with self-reported SARS-CoV-2 (OR 1.36; 95% CI 1.21–1.53). Analyses stratified by age found statistical evidence for an increase in risk in young adults (ages 18–29; OR 2.43, 95%CI = 1.82–3.23; n_self-harm behaviour_ = 530) and middle-aged people (ages 45–59 OR: 1.52, 95%CI = 1.25–1.84; n_self-harm behaviour_ = 1,329), but not in other age groups. These differences remained when models were adjusted for anxiety and depressive symptoms as well as for recent physical and/or psychological abuse.

Abel et al.^14^ analysed clinical codes from a large primary care database identifying 232,780 adults (≥16 years old) in the United Kingdom who had a positive polymerase chain reaction (PCR) test for SARS-CoV-2 (total sample n = 11,923,499). Their longitudinal survival analysis found an increased HR for self-harm in those with a positive PCR test (unadjusted HR 2.09; 95% CI 1.20-3.64); that association remained after adjustment for ethnicity, smoking, BMI, and physical health comorbidities (adjusted HR 2.21; 95% CI 1.11–4.39). Notably, the number of detected self-harm events in the study cohort was very low (SARS-CoV-2 n = 13; no SARS-CoV-2 n = 25).

Lastly, Mortier et al.^15^ examined suicide outcomes in 5,164 healthcare workers from 10 hospitals during the first wave of the pandemic in Spain. This study combined people with an active suicide plan and those who had carried out a suicide attempt because of the small sample size of the latter (n = 6). Nevertheless, for the purposes of this review, the authors provided an analysis limited only to those who had attempted suicide. While only 17.4% of the entire sample of healthcare workers had been infected with SARS-CoV-2, three (50%) of the suicide attempts occurred in the SARS-CoV-2 group. Specifically, the proportion of suicide attempts was greater in people hospitalized with COVID-19 compared to those with no history of SARS-CoV-2 (n = 1(1.8%) versus n = 3(0.07%); PR 25.84, 95%CI 2.73–244.58).There was no difference in the proportion of healthcare workers who had attempted suicide between those with SARS-CoV-2 infection/COVID-19 disease without a hospitalization compared to those with no history of SARS-CoV-2 (n = 2(0.24%) versus n = 3(0.07%); PR 3.36, 95%CI 0.56–20.12).

Of note, the follow-up period in these studies was relatively short (in the order of weeks). None characterized longer term associations or investigated suicide death as an outcome.

### Suicidal and/or Self-Harm Thoughts

A total of 11 studies examined suicidal and/or self-harm thoughts in eight distinct samples of people with SARS-CoV-2 compared with a SARS-CoV-2-negative comparator group (Table 2). These comprised four studies reporting on general population samples, three studies based on the same low-income sample, two studies of healthcare workers, and two studies of military veterans.

The two studies described in the previous section deriving data from the UCL Social Study also captured data on suicidal/self-harm thoughts. The first identified that a large proportion of those who had been infected with SARS-CoV-2 reported these thoughts in the pandemic's first month (33.4% vs. 17.8%; PR 1.88, 95%CI 1.54–2.29).^12^ The second study found an increased odds of suicidal/self-harm thoughts in those with SARS-CoV-2 over the pandemic's first year (OR 1.17; 95% CI 1.09–1.25).^13^ As was the case for self-harm behaviour, increases in suicidal/self-harm thoughts were observed in young adults (ages 18–29 OR: 1.26, 95% CI 1.06–1.50; n_suicidal/self−harm thoughts_ = 1,168) and middle-aged people (ages 45–59 OR 1.27, 95%CI 1.13–1.42; n_suicidal/self−harm thoughts_ = 4,069) but not in other age groups. These findings remained for the entire sample when models were adjusted for anxiety, depressive symptoms, and recent physical or psychological abuse.

Perlis et al.^16^ analyzed data from an online survey of n = 91,791 U.S. adults of whom 5,945 (6.5%) reported a prior clinician diagnosis of COVID-19 disease or positive laboratory test for SARS-CoV-2 from May 2020 to February 2021. They found that this group had higher mean scores on the suicidal/self-harm thoughts item of the Patient Health Questionnaire-9 (PHQ-9) compared to those without SARS-CoV-2/COVID-19 (mean(SD): 1.5(1.1) versus 0.99(1.1), *p* < 0.001); indeed, this clinically meaningful difference was the largest difference between groups on any of the PHQ-9 items. However, this specific analysis was not adjusted for potential demographic or clinical confounders. Raifman et al.^17^ examined suicidal/self-harm thoughts in 1,415 U.S. adults surveyed from March 31-April 13, 2020 and found a higher proportion of those with self-reported COVID-19 illness had suicidal/self-harm thoughts (66.7% vs. 15.9%; adjusted PR 3.5, 95%CI 1.9–6.4). The number of participants with COVID-19 was, however, small (n = 12).

Three studies reported data from a single cohort of 6,607 low and middle income (<$75,000 annual income) adults in the U.S.^18–20^ A very high proportion of those with a self-reported positive SARS-CoV-2 test reported suicidal and/or self-harm thoughts within the previous two weeks in comparison to those who tested negative (91% vs. 44%; PR = 2.05, 95%CI 1.94 −2.19) and in comparison to those who did not test (91% vs. 21%; PR = . 4.30, 95%CI 4.03–4.59).^19^ Notably, those who tested negative were also more likely to report suicidal/self-harm ideation compared to those who did not test (44% vs. 21%; PR 2.09, 95%CI 1.94–2.26). Multivariable logistic regression weighted according to U.S. census variables (demographic and geographical) and accounting for demographic, financial, and clinical differences between groups found that SARS-CoV-2 infection was independently associated with suicidal and/or self-harm thoughts OR 1.95 (95%CI 1.20–3.19).^18^

Mortier et al.,^15^ whose study of Spanish healthcare workers is described above, also examined suicidal thoughts. They found no statistical evidence of a difference although, there was a graded reduction in the proportion of people reporting suicidal thoughts among those who were hospitalized for COVID-19 disease compared to those with no history of SARS CoV-2 (12.6% vs. 7.9%; PR = 1.61, 95%CI = 0.80–3.24). They did however find more suicidal thoughts in those with SARS-CoV-2 infection/COVID-19 disease without hospitalization compared to those with no history of SARS-CoV-2 (10% vs. 7.9%; PR = 1.27, 95%CI = 1.01–1.59); note that this finding emerged from bivariate PR calculations conducted by us for the purposes of this review and was not apparent in the three-way comparison conducted in the study itself. There was no statistical evidence of an association in the study's multivariable logistic regression models with adjustment for demographic, clinical, and work-related factors.

Bruffaerts et al.^21^ also conducted a study of Belgian healthcare workers (n = 6,409). They surveyed physicians and clinical psychologists from four hospitals and examined suicidal thoughts and behaviours (STBs), covering mainly thoughts (death wish 3.6%; suicidal ideation 1.5%, suicide plan 1.0%) and suicide attempt 0% (attempt n = 2)). They found no association between being quarantined with SARS-CoV-2 infection and any of these outcomes. However, the odds of each ideation outcome were higher in people hospitalized for COVID-19 disease and there was a risk of any STB in the hospitalized group (OR 11.5, 95% CI 2.2–60.5).

Na et al.^22,23^ conducted two studies of suicidal/self-harm thoughts in U.S. military veterans. One used a cross-sectional design with 3,078 veterans of whom 233 reported a diagnosis of COVID-19.^22^ It found a higher proportion of those with SARS-CoV-2 who reported recent thoughts (12.0% vs. 7.6%; PR 1.58, 1.09–2.29). This study also examined correlates of recent suicidal/self-harm thoughts in those with SARS-CoV-2. It relied on self-report survey responses to several validated scales and ad hoc questions related to potential correlates. The second study of 661 veterans including 77 (11.6%) who had been infected with SARS-CoV-2 had a case-control design (comparing cases with and without recent suicidal/self-harm thoughts).^23^ Veterans with SARS-CoV-2 were more likely to report suicidal/self-harm thoughts (27.3% vs. 18.2%; PR 1.50, 95%CI, 1.00 to 2.25). This study further tested for interaction effects related to age and life purpose finding that suicidal/self-harm thoughts following SARS-CoV-2 infection were more likely in middle aged (45–59) and older (60 + ) persons and in veterans whose pre-pandemic purpose in life scores fell in the lowest quartile.

## Discussion

This review sought to summarize and synthesise the published evidence concerning the relationship between SARS-CoV-2 infection/COVID-19 disease and suicide/self-harm. The small number of relevant studies overall, and specifically a dearth of research focused on behavioural outcomes, limits our ability to draw firm conclusions. Nevertheless, most studies included in our review did find evidence of an association between SARS-CoV-2 infection and STBs, including the two large studies as part of the UCL COVID-19 Social Study demonstrating associations with suicidal and/or self-harm thoughts as well as self-harm behaviour after adjusting for potential confounders. Specifically, analyses of data from the UCL COVID-19 Social Study found that associations generally persisted even after adjusting for anxiety symptoms, depressive symptoms, and adversity experiences.^13^ Of course, it is possible for there to be residual confounding due to the unavailability of additional potential confounding factors.^26^

Studies also generally found a higher prevalence of suicidal and/or self-harm thoughts in people who had been infected with SARS-CoV-2 compared to those who had not. Suicidal/self-harm thoughts may be quite limited as proxies for subsequent suicidal/self-harm *behaviours*.^27^ Nevertheless, at minimum, these findings indicate that many people with SARS-CoV-2 experience substantial distress. Therefore, one could not rule out the possibility of an impact on suicide rates.

More research is needed to contextualize and augment the findings of this review. For example, a study of clinical presentations for self-harm in Wales, posted as a preprint after the timeframe of our review, showed an association between SARS-CoV-2 infection and self-harm.^28^ Specifically, a prior history of a self-harm presentation appeared to increase the risk of SARS-CoV-2 infection; in contrast, infection was not associated with subsequent self-harm.^28^ Emerging evidence suggests that SARS-CoV-2 infection may have a bidirectional relationship with psychiatric illness with each being a risk factor for occurrence of the other.^29^ Therefore, it may be that at least some of the observed associations in our review, specifically those that did not control for the presence of mental disorders, may be confounded since people with psychiatric illnesses are at higher risk for both SARS-CoV-2 infection and STBs.

We acknowledge that the associations identified in our review are plausibly confounded by unmeasured factors, and that the findings that our review has generated should be considered preliminary and in need of replication. Nevertheless, we may speculate that there are several possible mechanisms by which SARS-CoV-2 infection could potentially confer risk for STBs. These include (a) the virus's direct, acute effects on the brain,^30–32^ (b) the direct effects of the body's immune response on the brain,^30–32^ (c) the longer-term physiological and/or psychological effects of a chronic illness for those experiencing prolonged symptoms (i.e. “long COVID”),^33^ (d) fears about one's own death and/or infecting one's loved ones,^13^ (e) the social stigma of being infected with SARS-CoV-2 that exists in some communities,^34^ and (e) negative socioeconomic impacts (e.g. unpaid time off work).^2^

Viral infections like SARS-CoV-2 have physiological effects including direct effects on the brain as well as indirect effects related to the body's immune response; these can lead to neuropsychiatric symptoms.^30–32,35^ Prior research has found that seropositivity with influenza B was associated with a past history of suicide attempts,^36^ but, notably, there was no association with coronaviruses. Another study of the impact of common viral infections also showed no association with increased suicide attempts or deaths.^37^ However, a study of 40,469 patients infected with SARS-CoV-2 found that 22.5% had at least one neuropsychiatric manifestation, including anxiety symptoms, mood symptoms, sleep difficulties, and suicidal thoughts.^38^

A substantial proportion of people infected with SARS-CoV-2 are asymptomatic and the remainder experience symptoms ranging from mild to severe to life-threatening.^39,40^ If a positive association with suicide/self-harm truly exists, it seems plausible that the strength of association would vary greatly in line with the severity and/or duration of illness experienced. There was at least some indication in our review that more severe or symptomatic illness, as reflected by the need for hospitalization, may have been associated with increased risk.^19^ Furthermore, a study by Wang et al.^39^ which examined suicidal/self-harm thoughts in patients from 13 medical centres in Hubei, China, was excluded from our review due to the lack of a non-infected comparator group. However, it found that the presence of a fever was associated with suicidal/self-harm thoughts (OR 3.97; 95% CI 2.07–7.63).^41^ Future iterations of this living review will examine associations between more severe and prolonged cases of COVID-19 and STBs in due course.

Higher levels of depression have specifically been found in COVID-19 patients,^42^ and depression is a key risk factor for suicide.^43^ The role of the immune system as a potential mediator of suicide risk in the context of depression has been noted in previous studies,^44^ and COVID-19 patients have an over-activated immune system response.^42^ Specifically changes occur in expression levels of pro-inflammatory factors and serum T cells^42^ that could, in turn, increase the risk of depression. The degree to which such a process may mediate any associations with STBs is also an important area of future inquiry.

This review cannot quantify the potential relative contribution of physiological effects of SARS-CoV-2 infection versus ancillary psychological effects such as fear of death or social impact such as having to take leave from work. However, notably, while actual infection with SARS-CoV-2 was associated with risk of STBs in adults in the United Kingdom, fear of contracting it was not, aside from suicidal/self-harm thoughts in those aged 60 + .^13^

We should emphasize that the majority of participants in the studies included in our review would have been aware that they had contracted SARS-CoV-2 with many hospitalized due to physical sequelae of COVID-19 disease. That awareness and those experiences can provoke adverse psychological consequences in some people that might include STBs. By definition, those who have been infected with SARS-CoV-2 have been in proximal contact with at least one other person infected with the virus and often likely multiple people who were infected. Being around others who are ill or who die from COVID-19 may also have a psychological impact that could confer risk. This appears to have been observed, for example, by Na et al.,^22^ who found a higher risk of suicidal/self-harm thoughts in people with SARS-CoV-2 who lived with someone who was also infected.

There is also some emerging evidence that SARS-CoV-2 infected individuals are experiencing stigma and discrimination because of their illness sometimes even in the context of recovery.^45^ This is particularly worrisome given that stigma has been associated with poverty, social isolation, and delays in accessing healthcare.^45^ This underscores the need for efforts to combat stigma including providing the public with accurate scientific information to combat myths.

Studies using retrospective news reports to investigate suicide during the pandemic were excluded from this review due to the high potential of bias in these reports and lack of scientific rigour regarding SARS-CoV-2 exposure. Nevertheless, it is noteworthy that a number of those studies did find COVID-19 infection as a contributor in many suicide decedents or identified suicides among individuals who tested SARS-CoV-2 positive.^46–53^ Whether or not those anecdotal reports reflect a true association, such reports may be a reason for concern. They could have the potential to perpetuate stigma and could also contribute to social learning/an inadvertent message that suicidal and/or self-harm are a culturally accepted way of coping with COVID-19 infection.

Our review has important limitations. It included a relatively small number of studies examining different populations over different time periods with heterogeneous suicide and/or self-harm related outcomes as well as methods for measuring those outcomes. For these reasons, data were deemed to be unsuitable for meta-analysis. As higher quality evidence emerges, meta-analysis may become appropriate and would enhance future iterations of this review. The included studies themselves also had a number of limitations. We deemed relatively few to have a low risk of bias. Additionally, investigation of the relationship between suicide and/or self-harm thoughts and behaviour and SARS-CoV-2 infection was often a secondary outcome reported in the reviewed articles without explicit a priori hypotheses set down regarding the potential impact of being infected and related illness on these outcomes. There were only four studies with comparative data on behavioural outcomes. Of these, two derived from the same national study, one used clinical codes that may be likely to under-detect instances of suicide attempts or self-harm,^54^ and one had a relatively small sample size for examining suicide attempt outcomes. While most cross-sectional studies appeared to be measuring STBs after participants had been infected with SARS-CoV-2, this was often not explicitly demonstrated and therefore our review cannot draw conclusions about causality. Many of the studies also used ad hoc self-report measures which may or may not be reliable. As mentioned, most studies focused on suicidal and/or self-harm thoughts, which can be relatively poor proxies for behavioural outcomes and especially for death by suicide.^27^ Included studies often relied on self-report of STBs or clinical records which may both under-detect these phenomena. Some of the studies had non-random samples and/or did not run analyses to account for potential confounders. The potential for residual confounding for all of the results presented here is also substantial. For example, hospitalization itself can be a traumatic experience^55^ and therefore we cannot necessarily conclude that increased risk of STBs in those hospitalized for COVID-19 disease arose due to the disease itself rather than the experience of hospitalization. Importantly, studies included in our review focused on adults and therefore we cannot comment on any potential relationship (or lack thereof) between SARS-CoV-2 infection and STBs in children and adolescents. Likewise, there was a dearth of studies in lower- and middle-income countries. Different cultural norms and attitudes including stigma, more limited public health and healthcare system resources, higher infection rates and economic burden of the pandemic in these countries, might all be expected to have an influence on interactions between SARS-CoV-2 and STBs.

In summary, our review found some evidence of potential associations between SARS-CoV-2 infection and STBs both in the general population and in subpopulations (e.g., healthcare workers, military veterans). There were few studies examining behavioural outcomes and no studies, to date, examining suicide death. These preliminary findings suggest the need for more research in this area including studies conducted using large cohorts or samples, with prospective designs, explicitly aimed at examining death by suicide and non-fatal self-harm as outcomes, and from more diverse study populations including those of low- and middle-income countries. Our review provides at least some evidence that STBs may be an additional potential harmful consequence of SARS-CoV-2 infection. If that is ultimately confirmed, it would indicate a need for enhanced surveillance and prevention in those who have been infected including timely psychological support.

## Supplemental Material

sj-pdf-1-cpa-10.1177_07067437221094552 - Supplemental material for SARS-CoV-2 Infection and the Risk of Suicidal and Self-Harm Thoughts and Behaviour: A Systematic Review Click here for additional data file. Supplemental material, sj-pdf-1-cpa-10.1177_07067437221094552 for SARS-CoV-2 Infection and the Risk of Suicidal and Self-Harm Thoughts and Behaviour: A Systematic Review by Mark Sinyor, Rabia Zaheer, Roger T. Webb, Duleeka Knipe, Emily Eyles, Julian P.T. Higgins, Luke McGuinness, Lena Schmidt, Catherine Macleod-Hall, Dana Dekel, David Gunnell and Ann John in The Canadian Journal of Psychiatry

sj-docx-2-cpa-10.1177_07067437221094552 - Supplemental material for SARS-CoV-2 Infection and the Risk of Suicidal and Self-Harm Thoughts and Behaviour: A Systematic Review Click here for additional data file. Supplemental material, sj-docx-2-cpa-10.1177_07067437221094552 for SARS-CoV-2 Infection and the Risk of Suicidal and Self-Harm Thoughts and Behaviour: A Systematic Review by Mark Sinyor, Rabia Zaheer, Roger T. Webb, Duleeka Knipe, Emily Eyles, Julian P.T. Higgins, Luke McGuinness, Lena Schmidt, Catherine Macleod-Hall, Dana Dekel, David Gunnell and Ann John in The Canadian Journal of Psychiatry

sj-docx-3-cpa-10.1177_07067437221094552 - Supplemental material for SARS-CoV-2 Infection and the Risk of Suicidal and Self-Harm Thoughts and Behaviour: A Systematic Review Click here for additional data file. Supplemental material, sj-docx-3-cpa-10.1177_07067437221094552 for SARS-CoV-2 Infection and the Risk of Suicidal and Self-Harm Thoughts and Behaviour: A Systematic Review by Mark Sinyor, Rabia Zaheer, Roger T. Webb, Duleeka Knipe, Emily Eyles, Julian P.T. Higgins, Luke McGuinness, Lena Schmidt, Catherine Macleod-Hall, Dana Dekel, David Gunnell and Ann John in The Canadian Journal of Psychiatry

sj-docx-4-cpa-10.1177_07067437221094552 - Supplemental material for SARS-CoV-2 Infection and the Risk of Suicidal and Self-Harm Thoughts and Behaviour: A Systematic Review Click here for additional data file. Supplemental material, sj-docx-4-cpa-10.1177_07067437221094552 for SARS-CoV-2 Infection and the Risk of Suicidal and Self-Harm Thoughts and Behaviour: A Systematic Review by Mark Sinyor, Rabia Zaheer, Roger T. Webb, Duleeka Knipe, Emily Eyles, Julian P.T. Higgins, Luke McGuinness, Lena Schmidt, Catherine Macleod-Hall, Dana Dekel, David Gunnell and Ann John in The Canadian Journal of Psychiatry
