# Supplementary material for: SARS-CoV-2 Infection and the Risk of Suicidal and Self-Harm Thoughts and Behaviour: A Systematic Review
Source: Can J Psychiatry. 2022 May 9;67(11):813–28. doi: 10.1177/07067437221094552 (PMC9096003; doi:10.1177/07067437221094552)
Supplement: sj-pdf-1-cpa-10.1177_07067437221094552 - Supplemental material for SARS-CoV-2 Infection and the Risk of Suicidal and Self-Harm Thoughts and Behaviour: A Systematic Review [file sj-pdf-1-cpa-10.1177_07067437221094552.pdf]

Search strategies for "The impact of the COVID-19 pandemic on self-harm and suicidal behaviour: update of living systematic review" and associated publications

Scopus

TITLE-ABS-KEY("selfharm*" OR "self harm*" OR "self-harm*" OR "self injur*" OR "selfinjur*" OR "self-injur*" OR "selfmutilat*" OR "self mutilat*" OR "self-mutilat*" OR "suicid*" OR "parasuicid*" OR "suicide" OR "suicidal ideation" OR "attempt* suicide" OR "suicide attempt*" OR "drug overdose" OR "selfpoisoning" OR "self poisoning" OR "self-poisoning" OR "self-injurious behavi*" OR "selfmutilation" OR "self mutilation" OR "self-mutilation" OR "automutilation" OR "suicidal behavi*" OR "selfdestructive behavi*" OR "self destructive behavi*" OR "self-destructive behavi*" OR "selfimmolat*" OR "self-immolat*" OR "self immolat*" OR "cutt*" OR "headbang" OR "head-bang" OR "head bang" OR "overdose" OR "selfinflict*" OR "self-inflict*" OR "self inflict*" OR "hopelessness" OR "powerlessness" OR "helplessness" OR "negative attitude*" OR "emotional negativism" OR "pessimism" OR "depress*" OR "hopelessness depression" OR "passivity" OR "sad-affect" OR "sadness" OR "decreased affect" OR "cognitive rigidity" OR "suicidality" OR "suicide ideation") AND TITLE-ABS-KEY("nCoV" OR "HCoV" OR "covid 19" OR "covid-19" OR "covid19" OR "coronavirus" OR "19 ncov" OR "19-ncov" OR "2019 ncov" OR "2019-ncov" OR "2019ncov" OR "n-cov" OR "ncov" OR "coronavirus disease*" OR "sars-cov-2" OR "sars cov 2" OR "sars-cov 2" OR "mers-cov" OR "mers cov") AND PUBYEAR > 2020

- ➔ *The filter 'PUBYEAR > 2020' corresponds to the 2021 version of this search, in previous years we used 'PUBYEAR > 2018' and 'PUBYEAR > 2019' respectively*

Medline via PubMed

((mental health[TIAB] OR selfharm*[TIAB] OR self-harm*[TIAB] OR selfinjur*[TIAB] OR self-injur*[TIAB] OR selfmutilat*[TIAB] OR self-mutilat*[TIAB] OR suicid*[TIAB] OR parasuicid*[TIAB) OR (suicide[TIAB] OR suicidal ideation[TIAB] OR attempted suicide[TIAB]) OR (drug overdose[TIAB] OR self?poisoning[TIAB]) OR (self-injurious behavio?r[TIAB] OR self?mutilation[TIAB] OR automutilation[TIAB] OR suicidal behavio?r[TIAB] OR self?destructive behavio?r[TIAB] OR self?immolation[TIAB])) OR (cutt*[TIAB] OR head?bang[TIAB] OR overdose[TIAB] OR self?immolat*[TIAB] OR self?inflict*[TIAB]) OR (hopelessness[TIAB] OR powerlessness[TIAB] OR helplessness[TIAB] OR negative attitude$[TIAB] OR emotional negativism[TIAB] OR pessimism[TIAB] OR depress*[TIAB] OR hopelessness depression[TIAB] OR passivity[TIAB] OR sad-affect[TIAB] OR sadness[TIAB] OR decreased affect[TIAB] OR cognitive rigidity[TIAB] OR suicidality[TIAB] OR suicide ideation[TIAB]))) AND ((coronavirus disease?19[TIAB] OR sars?cov?2[TIAB] OR mers?cov[TIAB]) OR (19?ncov[TIAB] OR 2019?ncov[TIAB] OR n?cov[TIAB]) OR (\"severe acute respiratory syndrome coronavirus 2\" [Supplementary Concept] OR \"COVID-19\" [Supplementary Concept] OR COVID-19 [tw] OR COVID 2019 [tw] OR coronavirus [tw] OR nCoV[TIAB] OR HCoV))

Psy- and SocArXiv (both same query)

"(mental health OR selfharm* OR self-harm* OR selfinjur* OR self-injur* OR selfmutilat* OR self-mutilat* OR suicid* OR parasuicid* OR suicide OR suicidal ideation OR attempted suicide OR drug overdose OR self?poisoning OR self-injurious behavio?r OR self?mutilation OR automutilation OR suicidal behavio?r OR self?destructive behavio?r OR self?immolation OR cutt* OR head?bang OR overdose OR self?immolat* OR self?inflict* OR hopelessness OR powerlessness OR helplessness OR negative attitude OR emotional negativism OR pessimism OR depress* OR hopelessness depression OR passivity OR sad-affect OR sadness OR decreased affect OR cognitive rigidity OR suicidality OR suicide ideation) AND (coronavirus disease?19 OR sars?cov?2 OR mers?cov OR 19?ncov OR 2019?ncov OR n?cov OR COVID-19 OR COVID 2019 OR coronavirus OR nCoV OR HCoV)"

- ➔ *No date/content filters applied*

Med and BioRxiv, WHO Covid-19 database

We directly retrieve ALL new publications related to Covid-19 from these sources, see http://connect.biorxiv.org/relate/content/181 for Bio- MedRxiv Covid feed and https://search.bvsalud.org/global-literature-on-novel-coronavirus-2019-ncov/ for WHO on a daily basis. There is no date filter, we retrieve each new report as it becomes available.
We then apply our systematic search to those results, as described here:
McGuinness et al., (2020). medrxivr: Accessing and searching medRxiv and bioRxiv preprint data in R. Journal of Open Source Software, 5(54), 2651. https://doi.org/10.21105/joss.02651

The search strategy is below, please note that the syntax is RegularExpression, but it was designed to correspond to the PubMed query you see above, with new lines being joined with 'OR' statements. The only exception is the removal of the AND statement relating to COVID-19 itself, because those 3 sources only include COVID-specific information.

```
[Ss]elf[- ]?[Ii]njur(y|ious)[- ]?[Bb]ehaviou?r
[Ss]elf[- ]?([Mm]utilat¬[Ii]mmolat)(ion|ed)
[Aa]uto[- ]?[Mm]utilat(ion|ed)
[Ss]uicidal[- ]?[Bb]ehaviou?r
[Ss]elf[- ]?[Dd]estructive)[- ]?[Bb]ehaviou?r
[Ss]uicide
[Aa]ttempted[- ]?[Ss]uicide
[Ss]uicidal[- ]?[Ii]deation
[Ss]elf[- ]?[Hh]arm
[Ss]elf[- ]?[Mm]utilat
[Ss]elf[- ]?[Ii]njur
[Pp]ara[ -]?[Ss]uicid
[Dd]rug[ -]?[Oo]verdose
[Ss]elf[- ]?[Pp]oison(ing|ed)
```

[Ss]elf[-]?[iI]nflict
[Ss]elf[-]?[iI]mmolat
[Cc]utt
[Hh]ead[-]?[Bb]ang
[Oo]verdos
[Hh]opelessness
[Pp]owerlessness
[Hh]elplessness
[Nn]egative[-]?[Aa]ttitude
[Ee]motional[-]?[Nn]egativism
[Pp]essimism
[Dd]epress
[Pp]assivity
[Ss]ad[-]?[Aa]ffect
[Ss]adness
[Dd]ecreased[-]?[Aa]ffect
[Cc]ognitive[-]?[Rr]igidity
[Ss]uicidality
[Ss]uicide[-]?[Ii]deation
[Mm]ental[-]?[Hh]ealth
[Mm]ental[-]?[Hh]ealth[-]?([Cc]ris[ei]s|emergenc)
([pP]sychiatric|[Pp]sychotic|[Ss]chizophren\w*|[Bb]ipolar|[Mm]ental\w* ([Ii]ll\w*|[Dd]isorder))[-]?([Cc]ris[ie]s|[Ee]mergenc|[Aa]cute)
([Cc]ris[ie]s|[Ee]mergenc\w*|[Aa]cute)[-]?([pP]sychiatric|[Pp]sychotic|[Ss]chizophren\w*|[Bb]ipolar|[Mm]ental\w* ([Ii]ll\w*|[Dd]isorder))
