# Supplementary material for: SARS-CoV-2 Infection and the Risk of Suicidal and Self-Harm Thoughts and Behaviour: A Systematic Review
Source: Can J Psychiatry. 2022 May 9;67(11):813–28. doi: 10.1177/07067437221094552 (PMC9096003; doi:10.1177/07067437221094552)
Supplement: sj-docx-2-cpa-10.1177_07067437221094552 - Supplemental material for SARS-CoV-2 Infection and the Risk of Suicidal and Self-Harm Thoughts and Behaviour: A Systematic Review [file sj-docx-2-cpa-10.1177_07067437221094552.docx]

**Supplementary Appendix B: Summary of Non-Comparator Studies**

Only two non-controlled studies reported on suicidal and/or self-harm behaviour (Table 3). A case series of 50 COVID-19 patients who received a psychiatric consultation while hospitalized in Doha Qatar found three (6%) who reported a recent suicide attempt (Iqbal et al., 2020). A similar case series of 89 patients in Istanbul, Turkey found that the reason for consultation was a suicide attempt in one case although, notably, the study did not investigate whether suicide attempts may have been present in others in the sample (Turan et al., 2021).

Eight studies reported on suicidal and/or self-harm thoughts (Table 4). These included the two above in which 20% of the COVID-19 patients in Doha reported self-harm thoughts (Iqbal et al., 2020) and eight (9%) of the consultation requests in Istanbul were for suicidal thoughts (Turan et al., 2021).

A study of 460 COVID-19 patients from 13 medical centres in Hubei, China found that 23.3% of the total sample and 20.9% of the 187 included healthcare workers reported suicidal/self-harm thoughts in the prior two weeks (Wang et al., 2021). In the entire sample, the presence of fever was associated with these thoughts (OR 3.97; 95% CI, 2.07–7.63), as was female sex, lower education level, being divorced/widowed, and requiring psychological counselling. For healthcare workers only, lower education level and being divorced/widowed were associated.

A study of 77 COVID-19 patients (54 non-severe illness; 23 severe illness) in a hospital isolation ward in Wuhan, China found suicidal ideation in two people (2.6%) (He et al., 2021). One of these patients had non-severe COVID-19 illness and the other had severe illness. Another study of 106 COVID-19 patients in the isolation ward of a hospital in Huazhong, China found that 24.5% (26/106) reported suicidal/self-harm thoughts (Qian et al., 2020). A study of 370 people who survived COVID-19 in Wuhan found that 1.1% reported suicidal/self-harm thoughts (Wu et al., 2020). Three (9%) of 33 COVID-19 patients admitted to hospital in Gwangju, South Korea reported suicidal thoughts (Kim et al., 2020). Finally, 4.5% of people who had survived COVID-19 following an emergency presentation in Milan, Italy reported suicidal thoughts (Mazza et al., 2020).

**Supplementary Table 1: Suicide and/or Self-Harm Behavior in People with COVID-19 in Studies without a COVID-19-Negative Comparator Group**

| **Authors** | **Population and Setting** | **Study type** | **Exposure Measure** | **Sample size of SARS-CoV-2 Infected Individuals** | **COVID-19 status (U=Unclear, R=Recovered, A=Active)** | **Outcome Questionnaire Used** | **Suicidality Time span** | **Estimates/Findings** |
| --- | --- | --- | --- | --- | --- | --- | --- | --- |
| Iqbal et al., 2020 | Adult COVID-19 patients with a psychiatric consultation from three hospitals in Doha, Qatar | Case-series | PCR test | 50 | U | Electronic medical records | Not stated | 3 (6%) cases of non-fatal self-harm (each with a different method: overdose, self-laceration, and jumping from a height). Each of these cases was thought to be influenced by the psychosocial impact of the pandemic. One had a past history of psychiatric illness; Regarding symptoms of COVID-19 infection, two were asymptomatic and one had a mild case. |
| Turan et al., 2021 | Adult COVID-19  inpatients at Istanbul University-Cerrahpaşa, Cerrahpaşa Medical Faculty Hospital, Istanbul, Turkey who received psychiatric consultations  March 10, 2020 – June 26, 2020 | Case-series | “confirmed and probable cases" according to PCR test, chest imaging, and/or clinical criteria | 89 | A | Medical records | Not stated | 1 (1%) patient had a psychiatric consultation for a suicide attempt [note that this study only examined the reason for psychiatric consultation; it did not comment on the presence of suicide and/or self-harm behaviour in those who were referred/assessed for a different reason] |

**Supplementary Table 2: Suicide and/or Self-Harm Thoughts in People with COVID-19 in Studies without a COVID-19-Negative Comparator Group**

| **Authors** | **Population and Setting** | **Study type** | **Exposure Measure** | **Sample size of SARS-CoV-2 Infected Individuals** | **COVID-19 status (U=Unclear, R=Recovered, A=Active)** | **Outcome Questionnaire Used** | **Outcome Time span** | **Estimates/Findings** |
| --- | --- | --- | --- | --- | --- | --- | --- | --- |
| Wang et al., 2021 | COVID-19 patients with from 13 medical centres from Hubei, China  40.7% were Health Care Workers (HCWs)  February 2 - March 5, 2020 | Cross-sectional | Diagnosis in hospital including nucleic acid testing | 460 | A | PHQ-9 Item 9 | Past 2 weeks | 23.3% of the entire sample reported suicidal/self-harm thoughts and 2.83% reported severe (almost every day) thoughts  20.86% of HCWs reported self-mutilating or suicidal thoughts  Multiple logistic regression analysis  Higher odds of suicidal/self-harm thoughts were observed in female COVID-19 patients (OR = 1.97, 95% CI = 1.17–3.32), those who had undergone divorce or bereavement, (OR = 3.71, 95% CI = 1.52–9.01), patients with lower education levels (OR = 2.68, 95% CI = 1.66–4.33) those who had fever (OR=3.97, 95% CI = 2.07–7.63), and those who had psychological counselling (OR = 1.81, 95% CI = 1.07–3.05)  Multiple logistic regression analysis for HCWs  Higher odds of suicidal/self-harm thoughts were observed in patients with lower education levels (OR = 4.81, 95%CI = 1.41–16.43) and lower odds in those who were single (OR = 0.05, 95%CI = 0.01–0.40) or married (OR = 0.09, 95%CI = 0.02-0.59) compared to those who were divorced/bereaved |
| He et al., 2021 | COVID-19 patients admitted to the isolation ward of a hospital cancer center in Wuhan, China  February 14- March 14, 2020 | Cross-sectional | PCR test or serum SARS-CoV-2-specific IgM | 77 | A | Electronic medical record | Past 2 weeks | Suicidal ideation was identified in two (2.6%) patients of whom one non-severe COVID-19 illness and one had severe illness. |
| Mazza et al., 2020 | COVID-19 Survivors Adults Milan, Italy  April 6 to June 9, 2020 | Cross-sectional | Emergency department assessment including laboratory testing | 402 | R | Suicide item from the 13-item Beck Depression Inventory (BDI-13) | Not stated | 2.9% of COVID-19 survivors scored 1 (suicidal ideation), 0.8% scored 2 and 0.8% scored 3 (suicidal planning) on the BDI item. |
| Kim et al., 2020 | COVID-19 patients admitted to the Department of Infectious Diseases of Chonnam National University Bitgoeul Hospital (CNUBH), Gwangju, South Korea  February 29, - April 13, 2020. | Cross-sectional (data at baseline of an intervention trial) | Hospital diagnosis | 33 | A | Beck Depression Inventory (BDI) Item 9 | Not stated | 3 (9%) of patients reported suicidal ideation |
| Wu et al., 2020 | COVID‐19 survivors in Wuhan, China | Cross-sectional | Hospital diagnosis | 370 | U | PHQ-9 Item 9 | Past 2 weeks | 1.1% of survivors reported suicidal ideation |
| Iqbal et al., 2020 | Adult COVID-19 patients with a psychiatric consultation from three hospitals in Doha, Qatar | Case-series | PCR test | 50 | U | Electronic medical records | Not stated | 20% reported thoughts of self-harm. |
| Turan et al., 2021 | Adult inpatients at Istanbul University-Cerrahpaşa, Cerrahpaşa Medical Faculty Hospital, Istanbul, Turkey who received psychiatric consultations  March 10, 2020 – June 26, 2020 | Case-series | “confirmed and probable cases" according to PCR test, chest imaging, and/or clinical criteria | 89 | A | Medical records | Not stated | 9.0% of patients requested psychiatric consultation for suicidal ideation |
| Qian et al., 2021 | COVID-19 patients in the isolation ward of Tongji Hospital Affiliated to Tongji Medical College of Huazhong University of Science and Technology in China  February 2 -16, 2020 | Case-series | Hospital diagnosis | 106 | A | PHQ-9 Item 9 | Past 2 weeks | 24.53% (26/106) of patients had self-harm/suicidal ideation |

| **Supplementary Table 3: Quality Assessment of Cross-Sectional or Cohort Studies with a Comparator Group*** | | | | | | | | | | | | | | | | |  |
| --- | --- | --- | --- | --- | --- | --- | --- | --- | --- | --- | --- | --- | --- | --- | --- | --- | --- |
|  | 1. Was the research question or objective in this paper clearly stated? | 2. Was the study population clearly specified and defined? | 3. Was the participation rate of eligible persons at least 50%? | 4. Were all the subjects selected or recruited from the same or similar populations? | 5. Was a sample size justification, power description, or variance and effect estimates provided? | 6. For the analyses in this paper, were the exposure(s) of interest measured prior to the outcome(s) being measured? | 7. Was the timeframe sufficient so that one could reasonably expect to see an association between exposure and outcome if it existed? | 8. For exposures that can vary in amount or level, did the study examine different levels of the exposure as related to the outcome? | 9. Were the exposure measures clearly defined, valid, reliable, and implemented consistently across all study participants? | 10. Was the exposure(s) assessed more than once over time? | 11. Were the outcome measures clearly defined, valid, reliable, and implemented consistently across all study participants? | 12. Were the outcome assessors blinded to the exposure status of participants? | 13. Was loss to follow-up after baseline 20% or less? | 14. Were key potential confounding variables measured and adjusted statistically for their impact on the relationship between exposure(s) and outcome(s)? | | **Overall Rating** | |
| **Iob et al., 2020** | Y | Y | Y | Y | N | N | N | N | Yes | CD | Y | N/A | N/A | N | Fair | |  |
| **Sáiz et al., 2020^†^** | Y | Y | CD | Y | N | N | N | N | Y | N | N | N/A | N/A | N | Poor | |  |
| **Mortier et al., 2021** | Y | Y | N | Y | Y | N | N | Y | Y | N | Y | N/A | N/A | Y | Good | |  |
| **Bruffaerts et al., 2021** | Y | Y | N | Y | Y | N | N | Y | CD | N | Y | N/A | N/A | Y | Good | |  |
| **Elbogen et al., 2021^‡^** | Y | Y | Y | N | Y | N | N | N | Y | N | Y | N/A | N/A | Y | Fair | |  |
| **Tsai et al., 2021a^‡^** | Y | Y | Y | N | Y | N | N | N | Y | N | Y | N/A | N/A | Y | Fair | |  |
| **Tsai et al., 2021b^‡^** | Y | Y | Y | N | N | N | N | N | Y | N | Y | N/A | N/A | N | Fair | |  |
| **Paul & Fancourt, 2021** | Y | Y | Y | Y | Y | N | N | N | Y | N | Y | N/A | N/A | Y | Good | |  |
| **Ferrando et al., 2021^§^** | Y | Y | Y | Y | N | CD | CD | N | Y | N | Y | N | N/A | N | Poor | |  |
| **Perlis et., 2021** | Y | Y | CD | y | N | N | N | N | Y | N | Y | N/A | N/A | N | Fair | |  |
| **MiniguaN-Trujillo et al., 2021** | Y | Y | CD | Y | Y | N | N | N | Y | N | N | N/A | N/A | Y | Poor | |  |
| **Na et al., 2021a** | Y | Y | CD | Y | N | N | N | N | Y | N | Y | N/A | N/A | N | Fair | |  |
| **Auny et al., 2021** | Y | N | CD | CD | N | N | N | N | Y | N | N | N/A | N/A | N | Poor | |  |
| **Abel et al., 2021** | Y | Y | Y | Y | Y | Y | CD | N | Y | CD | Y | N | N | Y | Good | |  |
| **Raifman et al., 2020** | Y | Y | Y | Y | Y | N | N | N | Y | N | Y | N/A | N/A | Y | Good | |  |
| **Na et al., 2021b** | Y | Y | Y | Y | Y | N | N | N | Y | N | Y | N/A | N/A | Yes | Good | |  |
| N=No, Y=Yes, CD= Cannot Determine, N/A= Not Applicable  *NHLBI Study Quality tools used for assessment (available at: https://www.nhlbi.nih.gov/health-topics/studyquality-assessment-tools); note that, for the purposes of this review,  quality assessments focused on the quality of the methodology and presentation of data specifically related to the relationship between SARS-CoV-2 infection and suicide/self-harm  outcomes and not necessarily the overall study.  †Number of people tested for SARS-CoV-2 very low.  ‡ These three studies used the same data set - Results pooled in main text.  § Control group and SARS-CoV-2 group very different on socio-demographic and clinical factors, therefore risk of bias | | | | | | | | | | | | | | | | |  |

| **Supplementary Table 4: Quality Assessment of Cross-Sectional or Cohort Studies without a Comparator Group*** | | | | | | | | | | | | | | | | | | | | | | | | | | | | |
| --- | --- | --- | --- | --- | --- | --- | --- | --- | --- | --- | --- | --- | --- | --- | --- | --- | --- | --- | --- | --- | --- | --- | --- | --- | --- | --- | --- | --- |
|  | 1. Was the research question or objective in this paper clearly stated? | 2. Was the study population clearly specified and defined? | | 3. Was the participation rate of eligible persons at least 50%? | | 4. Were all the subjects selected or recruited from the same or similar populations? | | 5. Was a sample size justification, power description, or variance and effect estimates provided? | | 6. For the analyses in this paper, were the exposure(s) of interest measured prior to the outcome(s) being measured? | | 7. Was the timeframe sufficient so that one could reasonably expect to see an association between exposure and outcome if it existed? | | 8. For exposures that can vary in amount or level, did the study examine different levels of the exposure as related to the outcome? | | 9. Were the exposure measures clearly defined, valid, reliable, and implemented consistently across all study participants? | | 10. Was the exposure(s) assessed more than once over time? | | 11. Were the outcome measures clearly defined, valid, reliable, and implemented consistently across all study participants? | | 12. Were the outcome assessors blinded to the exposure status of participants? | | 13. Was loss to follow-up after baseline 20% or less? | | 14. Were key potential confounding variables measured and adjusted statistically for their impact on the relationship between exposure(s) and outcome(s)? | | **Overall Rating** |
| **Schwartz et al., 2021**^‡^ | Y | Y | Y | | Y | | N† | | N | | N | | N | | Y | | N | | N | | N/A | | N/A | | N | | Poor | |
| **Paz et al., 2020** | Y | Y | CD | | Y | | N | | N | | N | | N | | N | | N | | Y | | N/A | | N/A | | N | | Poor | |
| **Poyraz et al., 2021** | Y | Y | N | | Y | | N | | N | | N | | N | | Y | | N | | Y | | N | | N/A | | N | | Poor | |
| **Wang et al., 2021** | Y | Y | Y | | Y | | N | | N | | Y | | Y | | Y | | N | | Y | | N/A | | N/A | | Y | | Good | |
| **Kang et al., 2021** | Y | Y | Y | | Y | | N | | N | | Y | | N | | Y | | CD | | N | | N/A | | N/A | | N | | Poor | |
| **He et al., 2021** | Y | Y | CD | | Y | | N | | N | | N | | Y | | Y | | N | | Y | | N/A | | N/A | | N | | Fair | |
| **Mei et al., 2021** | Y | Y | Y | | Y | | N | | CD | | Y | | N | | Y | | CD | | N | | N | | Y | | N | | Poor | |
| **Mazza et al., 2020** | Y | Y | CD | | Y | | N | | Y | | Y | | N | | Y | | N | | Y | | N/A | | N/A | | N | | Fair | |
| **Fadipe et al., 2021**^§^ | Y | Y | CD | | Y | | N | | CD | | N | | N | | Y | | N | | N | | N/A | | N/A | | N | | Poor | |
| **Kim et al., 2020** | Y | Y | Y | | Y | | N | | Y | | CD | | N | | Y | | CD | | Y | | N/A | | N/A | | N | | Fair | |
| **Wu et al., 2020** | Y | Y | Y | | Y | | N | | Y | | CD | | N | | Y | | CD | | Y | | N/A | | N/A | | N | | Fair | |
| N=No, Y=Yes, CD= Cannot Determine, N/A= Not Applicable  *NHLBI Study Quality tools used for assessment (available at: https://www.nhlbi.nih.gov/health-topics/studyquality-assessment-tools); note that, for the purposes of this review, quality assessments focused on the quality of the methodology and presentation of data specifically related to the relationship between SARS-CoV-2 infection and suicide/self-harm outcomes and not necessarily the overall study.  † Note that lack of comparator data was considered a limitation in the quality assessment  ‡ Small Sample size limitation  § Eligibility criteria may introduce bias (only individuals with a smartphone eligible) | | | | | | | | | | | | | | | | | | | | | | | | | | | | |

| **Supplementary Table 5: Quality Assessment of Case Series*** | | | | | | | | | | |
| --- | --- | --- | --- | --- | --- | --- | --- | --- | --- | --- |
|  | 1. Was the study question or objective clearly stated? | 2. Was the study population clearly and fully described, including a case definition? | 3. Were the cases consecutive? | 4. Were the subjects comparable? | 5. Was the intervention clearly described? | 6. Were the outcome measures clearly defined, valid, reliable, and implemented consistently across all study participants? | 7. Was the length of follow-up adequate? | 8. Were the statistical methods well-described? | 9. Were the results well-described? | **Overall Rating** |
| **Nalleballe et al., 2020** | Y | CD | CD | CD | N/A | N | N/A | N/A | Y | Poor |
| **Iqbal et al., 2020** | Y | Y | Y | Y | N/A | Y | N/A | N/A | Y | Good |
| **Turan et al.,2021** | Y | Y | Y | Y | N/A | CD | N/A | N/A | Y | Fair |
| **Qian et al., 2020** | Y | Y | Y | Y | N/A | Y | N/A | N/A | Y | Good |
| N=No, Y=Yes, CD= Cannot Determine, N/A= Not Applicable  *NHLBI Study Quality tools used for assessment (available at: https://www.nhlbi.nih.gov/health-topics/studyquality-assessment-tools); note that, for the purposes of this review, quality assessments focused on the quality of the methodology and presentation of data specifically related to the relationship between SARS-CoV-2 infection and suicide/self-harm outcomes and not necessarily the overall study. | | | | | | | | | | |

**Supplementary References**

1. Iob E, Steptoe A, Fancourt D. Abuse, self-harm and suicidal ideation in the UK during the COVID-19 pandemic. The British Journal of Psychiatry 2020;217(4):543–546.
2. Sáiz PA, de la Fuente-Tomas L, García-Alvarez L, et al. Prevalence of Passive Suicidal Ideation in the Early Stage of the Coronavirus Disease 2019 (COVID-19) Pandemic and Lockdown in a Large Spanish Sample. J Clin Psychiatry 2020;81(6):20l13421.
3. Mortier P, Vilagut G, Ferrer M, et al. Thirty-day suicidal thoughts and behaviors among hospital workers during the first wave of the Spain COVID-19 outbreak. Depress Anxiety 2021;528–544.
4. Bruffaerts, R., Voorspoels, W., Jansen, L., Kessler, R. C., Mortier, P., Vilagut, G., De Vocht, J., & Alonso, J. (2021). Suicidality among healthcare professionals during the first COVID19 wave. Journal of affective disorders, 283, 66–70. <https://doi.org/10.1016/j.jad.2021.01.013>
5. Elbogen EB, Lanier M, Blakey SM, et al. Suicidal ideation and thoughts of self-harm during the COVID-19 pandemic: The role of COVID-19-related stress, social isolation, and financial strain. Depress Anxiety. Epub ahead of print May 5, 2021. DOI: 10.1002/da.23162.
6. Tsai J, Elbogen EB, Huang M, et al. Psychological distress and alcohol use disorder during the COVID-19 era among middle- and low-income U.S. adults. J Affect Disord 2021a;28841–49.
7. Tsai J, Huang M, Elbogen E. Mental Health and Psychosocial Characteristics Associated With COVID-19 Among U.S. Adults. Psychiatr Serv 2021b;72(4):444–447.
8. Paul E, Fancourt D. Factors influencing self-harm thoughts and behaviours over the first year of the COVID-19 pandemic in the UK: longitudinal analysis of 49 324 adults. The British Journal of Psychiatry 2021;1–7.
9. Ferrando SJ, Klepacz L, Lynch S, et al. Psychiatric emergencies during the height of the COVID-19 pandemic in the suburban New York City area. J Psychiatr Res 2021;136552–559.
10. Perlis RH, Santillana M, Ognyanova K, et al. Factors Associated With Self-reported Symptoms of Depression Among Adults With and Without a Previous COVID-19 Diagnosis. JAMA Netw Open 2021;4(6):e2116612.
11. Miniguano-Trujillo A, Salazar F, Torres R, et al. An integer programming model to assign patients based on mental health impact for tele-psychotherapy intervention during the Covid–19 emergency. Health Care Manag Sci 2021;24(2):286–304.
12. Na P, Tsai J, Harpaz-Rotem I, et al. Mental health and suicidal ideation in US military veterans with histories of COVID-19 infection. BMJ Mil Health 2021a;bmjmilitary-2021-001846.
13. Auny FM, Akter T, Guo T, et al. How Has the COVID-19 Pandemic Changed BMI Status and Physical Activity – Its Associations with Mental Health Conditions, Suicidality: An Exploratory Study. Risk Manag Healthc Policy 2021;142527–2536. 1.
14. Abel KM, MJ, Ashcroft DM, et al. COVID-19 infection and subsequent psychiatric morbidity, sleep problems and fatigue: analysis of an English primary care cohort of 226,521 positive patients.
15. Raifman J, Ettman CK, Dean L, et al. Economic precarity, social isolation, and suicidal ideation during the COVID-19 pandemic.
16. Na PJ, Tsai J, Hill ML, et al. Prevalence, risk and protective factors associated with suicidal ideation during the COVID-19 pandemic in U.S. military veterans with pre-existing psychiatric conditions. J Psychiatr Res 2021b;137351–359.Schwartz DA, Connerney MA, Davila-Molina M, et al. Resident Mental Health at the Epicenter of the COVID-19 Pandemic. Acad Med 2020;10.1097/ACM.0000000000003768.
17. Schwartz DA, Connerney MA, Davila-Molina M, et al. Resident Mental Health at the Epicenter of the COVID-19 Pandemic. Acad Med 2020;10.1097/ACM.0000000000003768.
18. Paz C, Mascialino G, Adana-Díaz L, et al. Behavioral and sociodemographic predictors of anxiety and depression in patients under epidemiological surveillance for COVID-19 in Ecuador. PLOS ONE. 2020;15(9):e0240008.
19. Poyraz BÇ, Poyraz CA, Olgun Y, et al. Psychiatric morbidity and protracted symptoms after COVID-19. Psychiatry Res 2021;295113604.
20. Wang, M., Hu, C., Zhao, Q. et al. Acute psychological impact on COVID-19 patients in Hubei: a multicenter observational study. Transl Psychiatry 11, 133 (2021). https://doi.org/10.1038/s41398-021-01259-0
21. Kang E, Lee SY, Kim MS, et al. The Psychological Burden of COVID-19 Stigma: Evaluation of the Mental Health of Isolated Mild Condition COVID-19 Patients. J Korean Med Sci 2021;36(3):e33.
22. He X, Zhang D, Zhang L, et al. Neurological and psychiatric presentations associated with COVID-19. Eur Arch Psychiatry Clin Neurosci. Epub ahead of print March 12, 2021. DOI: 10.1007/s00406-021-01244-0.
23. Mei Q, Wang F, Bryant A, et al. Mental health problems among COVID‐19 survivors in Wuhan, China. World Psychiatry 2021;20(1):139–140.
24. Mazza MG, De Lorenzo R, Conte C, et al. Anxiety and depression in COVID-19 survivors: Role of inflammatory and clinical predictors. Brain Behav Immun 2020;89594–600.
25. Fadipe B, Oshodi YO, Umeh C, et al. Psychosocial health effects of Covid-19 infection on persons in treatment centers in Lagos, Nigeria. Brain Behav Immun Health 2021;16100284.
26. Kim J-W, Stewart R, Kang S-J, et al. Telephone based Interventions for Psychological Problems in Hospital Isolated Patients with COVID-19. Clin Psychopharmacol Neurosci 2020;18(4):616–620.
27. Wu C, Hu X, Song J, et al. Mental health status and related influencing factors of COVID‐19 survivors in Wuhan, China. Clin Transl Med 2020;10(2):e52.
28. Nalleballe K, Reddy Onteddu S, Sharma R, et al. Spectrum of neuropsychiatric manifestations in COVID-19. Brain Behav Immun 2020;8871–74.
29. Iqbal Y, Al Abdulla MA, Albrahim S, et al. Psychiatric presentation of patients with acute SARS-CoV-2 infection: a retrospective review of 50 consecutive patients seen by a consultation-liaison psychiatry team. BJPsych Open 2020;6(5):e109.
30. Turan Ş, Poyraz BÇ, Aksoy Poyraz C, et al. Characteristics and outcomes of COVID-19 inpatients who underwent psychiatric consultations. Asian J Psychiatr 2021;57102563.
31. Qian Z, Caihong H, Renjie F, et al. Anxiety, depression and physical symptoms in patients with novel coronavirus pneumonia [J] . Chin. J. Neurol 2020 ;53(06):432-436. DOI: 10.3760/cma .j.cn113694-20200220-00102
