# Supplementary material for: SARS-CoV-2 Infection and the Risk of Suicidal and Self-Harm Thoughts and Behaviour: A Systematic Review
Source: Can J Psychiatry. 2022 May 9;67(11):813–28. doi: 10.1177/07067437221094552 (PMC9096003; doi:10.1177/07067437221094552)
Supplement: sj-docx-4-cpa-10.1177_07067437221094552 - Supplemental material for SARS-CoV-2 Infection and the Risk of Suicidal and Self-Harm Thoughts and Behaviour: A Systematic Review [file sj-docx-4-cpa-10.1177_07067437221094552.docx]

| **Supplementary Appendix C** | |
| --- | --- |
| Study | Funding Statements |
| Iob et al., 2020^11^ | “This COVID-19 Social Study was funded by the Nuffield Foundation (WEL/FR-000022583), but the views expressed are those of the authors and not necessarily the Foundation. The study was also supported by the MARCH Mental Health Network, funded by the Cross-Disciplinary Mental Health Network Plus initiative supported by UK Research and Innovation (ES/S002588/1), and by the Wellcome Trust (221400/Z/20/Z). D.F. was funded by the Wellcome Trust (205407/Z/16/Z). The funders had no final role in the study design; in the collection, analysis and interpretation of data; in the writing of the report; or in the decision to submit the paper for publication. All researchers listed as authors are independent of the funders and all final decisions about the research were taken by the investigators and were unrestricted.” |
| Paul & Fancourt, 2021^12^ | “This work was supported by the Nuffield Foundation (D.F.: WEL/FR-000022583), the MARCH Mental Health Network funded by the Cross-Disciplinary Mental Health Network Plus initiative supported by UK Research and Innovation (D.F.: ES/S002588/1), and the Wellcome Trust (D.F.: 221400/Z/20/Z and 205407/Z/16/Z).” |
| Abel et al., 2021*^13^ | “This study was conducted using data from the CPRD obtained under licence from the UK Medicines and Healthcare products Regulatory Agency (MHRA). The data are provided by patients and collected by the NHS as part of their care and support. The interpretation and conclusions contained in this study are those of the authors alone, and not necessarily those of the MHRA, NHS, NIHR, or the UK Department of Health and Social Care. We would like to acknowledge all the data providers and general practices that made the anonymised data available for research. The study was approved by the Independent Scientific Advisory Committee for CPRD research (20_094R2).” |
| Perlis et al., 2021^15^ | “This study was supported by the National Institute of Mental Health (grant R01MH116270 to Dr Perlis), the Blyth Family Fund, and the National Science Foundation (support to Drs Ognyanova, Lazar, and Baum).” |
| Raifman et al., 2020*^16^ | “This study was funded in part through support from the Rockefeller Foundation Boston University 3-D Commission. Ms Ettman worked on this project while funded by grant No. T32 AG 23482 15 from the National Institutes of Health. Dr. Raifman worked on this project while funded by K01 MH116817.” |
| Elbogen et al., (2021)**^17^ | “This study was supported with internal university funds by the University of Texas Health Science Center at Houston. The views expressed in this article are those of the authors and do not necessarily reflect the position or policy of any University or of the United States Government or Department of Veterans Affairs (VA). Dr. Blakey was supported by a VA Office of Academic Affiliations Advanced Fellowship in Mental Illness Research and Treatment.” |
| Tsai et al., (2021)**^18^ | “Financial support was provided by internal university funds from the University of Texas Health Science Center at Houston.” |
| Tsai et al., (2021)**^19^ | “This study was supported by internal funds from the University of Texas Health Science Center at Houston.” |
| Mortier et al., 2021^14^ | “This study was supported by Fondo de Investigación Sanitaria, Instituto de Salud Carlos III (Ministerio de Ciencia e Innovación)/FEDER (COV20/00711); ISCIII (Sara Borrell, CD18/00049) (PM); FPU (FPU15/05728) (LB); ISCIII (PFIS, FI18/00012) (BP); Generalitat de Catalunya (2017SGR452).” |
| Bruffaerts et al., 2021^20^ | “Drs. Jordi Alonso and Philippe Mortier reported funding for this study from ISCIII/FEDER (grant #COV20/00,711, JA) and ISCIII Sara Borrell (grant #CD18/00,049, PM). Dr. Ronny Bruffaerts reported funding from the Special Research Fund KULeuven (EDC-D9624-DOA/2020/007).” |
| Na et al., 2021^21^ | “This study was funded internally through the National Center for PTSD and thus does not have a grant number.” |
| Na et al., 2021^22^ | “The National Health and Resilience in Veterans Study is supported by the U.S. Department of Veterans Affairs National Center for Posttraumatic Stress Disorder.” |
